# Field Evaluation of N95 Filtering Facepiece Respirators on Construction Jobsites for Protection against Airborne Ultrafine Particles

**DOI:** 10.3390/ijerph15091958

**Published:** 2018-09-07

**Authors:** Atin Adhikari, Aniruddha Mitra, Abbas Rashidi, Imaobong Ekpo, Jacob Schwartz, Jefferson Doehling

**Affiliations:** 1 Department of Epidemiology & Environmental Health Sciences, Jiann-Ping Hsu College of Public Health, Georgia Southern University, Statesboro, GA 30460, USA; ie00146@georgiasouthern.edu; 2 Department of Mechanical Engineering, Georgia Southern University, Statesboro, GA 30460, USA; mitra@georgiasouthern.edu (A.M.); jd06011@georgiasouthern.edu (J.D.); 3 Department of Civil and Environmental Engineering, University of Utah, Salt Lake City, UT 84112, USA; abbas.rashidi@utah.edu; 4 Department of Manufacturing Engineering, Georgia Southern University, Statesboro, GA 30460, USA; js17203@georgiasouthern.edu

**Keywords:** aerosol, nanoparticle, ultrafine particles, masks, construction workers, occupational safety, industrial hygiene, N95 filtering facepiece respirators

## Abstract

Exposure to high concentrations of airborne ultrafine particles in construction jobsites may play an important role in the adverse health effects among construction workers, therefore adequate respiratory protection is required. The performance of particulate respirators has never been evaluated in field conditions against ultrafine particles on construction jobsites. In this study, respiratory protection levels against ultrafine particles of different size ranges were assessed during three common construction related jobs using a manikin-based set-up at 85 L/min air flow rate. Two NanoScan SMPS nanoparticle counters were utilized for measuring ultrafine particles in two sampling lines of the test filtering facepiece respirator—one from inside the respirator and one from outside the respirator. Particle size distributions were characterized using the NanoScan data collected from outside of the respirator. Two models of N95 respirators were tested—foldable and pleated. Collected data indicate that penetration of all categories of ultrafine particles can exceed 5% and smaller ultrafine particles of <36.5 nm size generally penetrated least. Foldable N95 filtering facepiece respirators were found to be less efficient than pleated N95 respirators in filtering nanoparticles mostly at the soil moving site and the wooden building frameworks construction site. Upon charge neutralization by isopropanol treatment, the ultrafine particles of larger sizes penetrated more compared to particles of smaller sizes. Our findings, therefore, indicate that N95 filtering facepiece respirators may not provide desirable 95% protection for most categories of ultrafine particles and generally, 95% protection is achievable for smaller particles of 11.5 to 20.5 nm sizes. We also conclude that foldable N95 respirators are less efficient than pleated N95 respirators in filtering ultrafine particles, mostly in the soil moving site and the wooden building framework construction site.

## 1. Introduction

Respirable airborne particles are commonly generated during various construction activities, including blasting, cutting, grinding, chipping, and drilling of concrete and wood. Moving soil and demolishing concrete structures and buildings also create respirable airborne particles. Also, a portion of the particles released during concrete cutting are respirable airborne particles composed of quartz [1,2]. A significant portion of the concentrations of respirable airborne particles (numbers of particles in certain air volume) could be ultrafine particles including particles of <100 nm sizes [3,4]. Based on the US National Nanotechnology Initiative (NNI) definition of nano dimensions [5], and ASTM E2456-06 standard [6], we may consider these <100 nm particles as nanoparticles as a sub category of ultrafine particles. Different industrial work processes may produce particles that have dimensions in the nanometer size range, which are often referred to as ultrafine particles [3]. We will use the term ultrafine particles in this article to avoid any confusion. A previous laboratory-based study conducted by the National Institute for Occupational Safety and Health (NIOSH) researchers demonstrated the potential generation of nanoparticles and other particles of 3–500 nm sizes during simulated grinding activity [7]. These observations warrant further study on exposure assessment of ultrafine particle in construction jobsites because, according to the March 2018 U.S. Bureau of Labor Statistics, approximately 7.15 million workers are employed in the U.S. construction industry [8].

Ultrafine particles are respirable deeper into our alveoli, beyond our body’s natural respiratory cleaning mechanisms such as cilia and mucous membranes, and are likely to be retained in the lower airways [9]. These particles have the potential to cause serious diseases, such as respiratory symptoms, lung cancer, and silicosis, depending on the components of the particles [10]. Respirable fine and ultrafine particles at construction sites may carry crystalline and amorphous silica and may stimulate and suppress the immune system and cause injury to cells of several organs [11,12,13,14,15,16]. The ultrafine particles are thought to have different toxic effects depending on their surface characteristics [17]. Recent studies suggest that the high surface area of silica and TiO_2_ nanoparticles may aggravate airway inflammation and may have adverse effects on human health [18,19]. On these premises, adequate respiratory protection by personal protective equipment like filtering facepiece respirators is relevant across varying construction tasks performed by construction workers.

The exposure levels of ultrafine particles through the above-described construction tasks are largely unknown, and previous studies mostly focused on coarse airborne particles, PM_2.5_, and PM_10_ (particles that have aerodynamic diameters less than or equal to 10.0 µm and 2.5 μm, respectively) [20]. Understanding the exposure levels and penetration levels of ultrafine particles through N95 masks, however, are critically important because recent laboratory studies have demonstrated cytotoxicity of silica nanoparticles on lung epithelial cells [21]. Furthermore, because nanoparticles have a much greater surface area [22], they can adsorb airborne NO_2_, SO_2_, and other pollutants [23] released from machines on construction jobsites. 

In general, it is believed that the dust generated during mechanical processes on construction jobsites is mostly coarse particles (>1 µm) formed through construction tasks described above, and this belief is in line with findings from previous studies [3,4]. Consequently, little attention has been paid to the ultrafine particles generated from these tasks and their exposure levels. These data are, however, essential because recent laboratory studies have suggested adverse health effects of ultrafine and fine particles, as described above.

Concrete grinding and cutting are very dusty jobs in the construction industry, which may pose a severe health risk to masons [24]. Masonry bricks, blocks, and concrete slabs may contain significant amounts of crystalline silica, which can be released into the workers’ breathing zone when these materials are dry-cut [24]. The exposure levels are often above [25] the Occupational Safety and Health Administration’s (OSHA) recently revised Permissible Exposure Limit (PEL) for silica, which is 50 µg/m^3^ [26], and Threshold Limit Values (TLV) of the American Conference of Government Industrial Hygienist (ACGIH) for crystalline silica, which is 25 µg/m^3^ for time-weighted average (TWA) of a 8 h work shift [27]. Therefore, continuous chronic exposure to these hazardous dust levels can lead to the development of silicosis among workers.

Building construction in the United States involves numerous wooden structures, which are routinely used in framing walls, floors, stairs, and landings in new building construction [28]. Numerous workers are involved in timber-based construction and wood dust is one of their most common occupational exposures [29]. Exposure to wood dust can be associated with a variety of adverse health effects among workers, such as dermatitis, allergic respiratory effects, mucosal and nonallergic respiratory effects, and occupational asthma [30]. The amount and size of particles released as wood dust differ according to the operations performed [31].

Engineering controls, effective respiratory protection, and work practices—when maximized—are valuable in preventing exposure to ultrafine particles [24]. In this exploratory research study, we evaluated simulated workplace protection factors offered by NIOSH approved N95 particulate filtering facepiece respirators against airborne ultrafine particles. Respiratory protection appears to be the most widely used preventive measure in the construction industry [32]. When performing heavy labor, however, it is often inconvenient for workers to work with respirators worn and their effectiveness might be questioned due to faceseal leakage [33,34]. The respirators provide insufficient protection when the protection factor is too low for a specific situation and when not used or appropriately maintained. The performance of particulate respirators generally used by construction workers (commonly N-series particulate respirators) was never evaluated in field conditions against ultrafine particles. Currently, there are concerns about protection against engineered nanoparticles. However, at this time there are no enforceable U.S. exposure limits for engineered nanoparticles. Furthermore, the performances of filtering facepiece respirators on real construction jobsites can be significantly different from results in laboratory conditions because of the: (a) Loading of dust particles on mask surfaces that may change pressure drop and affect penetration, and (b) ambient charged particles settled on surfaces of masks that may interfere with the filtration efficiency of ultrafine particles.

A previous NIOSH study [35] showed that the filtration media in N95 and P100 respirators can capture nanoparticles at acceptable levels, but also that in leakage tests, nanoparticles are able to differentially enter respirators in higher numbers. Researchers have also shown that there are marked differences in filtration efficiency among specific brands of N95 respirators. In another study from the same research group [36], the authors found that the shift in the most penetrating particle size from 45 to 150 nm for respirator filters with charge removed indicates that mechanical filters without charge may perform better against nanoparticles than electrostatic filters rated for the same filter efficiency. These laboratory studies, however, were conducted using NaCl particles. The efficiency of charge-removed N95 respirators to provide protection against ultrafine particles on construction jobsites is currently unknown. To address this, we examined the filtration efficiency of pleated and foldable models of N95 respirators, as described below.

Because the filtration mechanism for N95 respirators relies on electrical charges, charged particles in ambient air in construction can affect the charge of the respirators, which in turn may affect the capture of nanoparticles. To better understand the interaction between filtration without interference from electrostatic charge, we also conducted a few measurements where respiratory protection against nanoparticles was tested for N95 masks that have their electrostatic charge removed by isopropanol immersion, as described previously [36].

The primary objective of this exploratory study was to evaluate the filtration efficiency of N95 respirators with previously reported highest and lowest fit test rates against airborne ultrafine particles at construction jobsites by simultaneously measuring ultrafine particle levels inside and outside of the respirators fitted onto a manikin.

## 2. Materials and Methods

### 2.1. Construction Sites Selected for the Field Study and Descriptions of Sampling Methods

To assess N95 respirator penetration percentages of ultrafine particles at field conditions, three construction jobsites were selected. We targeted three common construction related tasks: (1) Concrete blasting and grinding; (2) wood cutting and other tasks during framing of wooden side walls, inner partition walls, and landings in a new building construction site; and (3) soil moving by bulldozers in a large construction site. Ultrafine particles in construction worksites—near the respirator evaluation system (outside the respirator and also from the inside of the respirators)—were measured by two SMPS (Scanning Mobility Particle Sizer) nanoparticle counters (Model 3910; TSI, Inc., Shoreview, MN, USA). Four key components of the NanoScan SMPS include 1. Pre-conditioner: The preconditioner cyclone can effectively remove larger particles, which is beneficial for sampling of ultrafine particles in construction environments contaminated with coarse dust particles. Two. Particle Charger: The unipolar charger in the instrument charges more nanoparticles than bipolar chargers without any radioactive material, which allowed us to estimate very low levels of nanoparticles, particularly inside the respirator mask. Three. Size Selector: A Radial DMA (RDMA) is available for size resolution and accuracy. Nanoparticle sizes ranging from 11.5 to 365 nm are detectable in this instrument in 13 size channels in 13 logarithmically-spaced size bins. Four. Particle Counter: An isopropanol-based condensation particle counter provides accurate measurements at high and low concentrations using a working fluid acceptable in workplace environments. NanoScan Manager Software (available from TSI, Inc., Shoreview, MN, USA) was used for the analysis of nanoparticle sizes and concentration levels.

**Figure 1:**
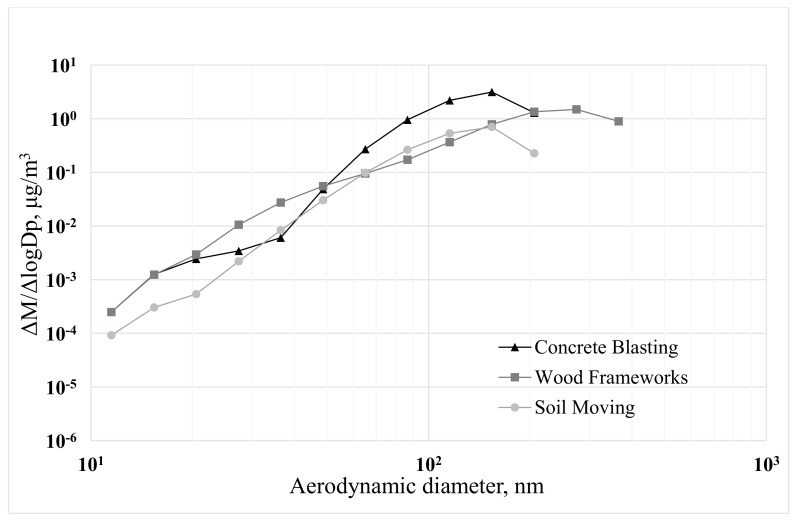
Size distributions of ultrafine particles at three different construction sites during the experiments with pleated N95 filtering facepiece respirators.

**Figure 2:**
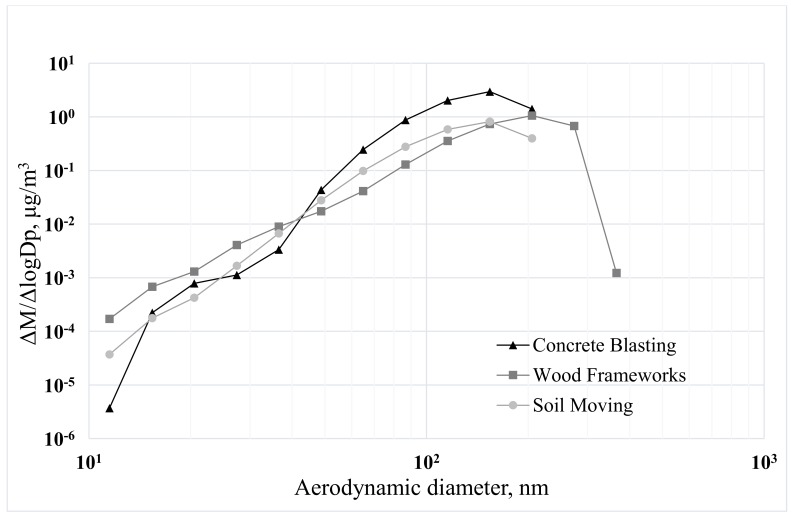
Size distributions of ultrafine particles at three different construction sites during the experiments with foldable N95 filtering facepiece respirators.

We analyzed size distributions of particles and mass concentrations based on the data collected outside the test N95 respirators (Figure 1 and Figure 2). Size distributions of ultrafine particles appear very stable despite the variations in construction activities in different locations.

When using two monitors in parallel, we found some discrepancies between the results obtained from the two NanoScan monitors, although both were of same models. Extensive calibration data revealed that one NanoScan was consistently measuring 75% of the other one over a wide spectrum of the particle sizes from the same location when running in parallel. Literature also shows that previous researchers encountered similar shortcomings [37,38,39], where the variations were as high as 30% between the similar particle monitors. Investigators discussed this issue in detail with the manufacturer, TSI, Inc. Based on the email communications [40], even a “calibrated” instrument can have ±20% error. Keeping in mind that this result will be used for comparative study, the authors considered a “middle ground” without violating the ±20% error limit for individual instrument. This “middle ground” would be 87.5%, an average between the 100% and 75%. The instrument with higher reading was consistently used outside the mask and its reading was reduced by 12.5%, so that it will read 87.5% instead of 100%. On the other hand, the instrument with lower reading was consistently used inside the mask and its reading was increased by 16.67%. A 16.67% increase in 75% will result in 87.5%. Based on this calibrated normalized data, both monitors provided the reading closer to actual number concentration of airborne particles. This fixed orientation of two NanoScans in our respirator evaluation set-up may create some bias overestimating the penetration percentage level. However, considering the main purpose of the study—evaluation of the simulated workplace protection factors provided by N95 respirators against ultrafine particles—researchers focused on the higher end of particle concentrations rather than underestimating the penetration level.

### 2.2. Assessment of Filtration Efficiency (Simulated Protection Factor) of N95 Filtering Facepiece Respirators against Airborne Nanoparticles at Field Conditions during Construction Works

In this specific task, we used two NanoScan SMPS nanoparticle counters to monitor nanoparticle levels in two sampling lines as shown in Figure 1—inside (Sampling probe—In) and outside (Sampling probe—Out) of an N95 respirator. The experimental set-up including manikin fitted with N95 respirator, two sampling lines connected with two NanoScan monitors, air sampling pump (adjusted to 85 L/min air flow rate)—all fixed on a revolving cart—was placed within five to ten meters from the actual work locations. The two sampling line tubes had the same length and diameters to obtain the same retention time and the same losses for collected particles. In this experiment, we examined protection levels offered by N95 disposable particulate respirator against ultrafine particles at three different construction jobsites, as described above.

Two types of NIOSH-approved N95 masks were tested in all experiments: (1) Pleated N95 mask and foldable N95 mask. We considered these two models because previous studies showed that similar N95 masks from two manufacturers showed different respiratory protection levels. For example, AOSafety Pleats Plus (TC 84A-2630, Aearo Corporation) previously showed highest fit test rate [41], whereas Model FR200 Affinity Folderable (TC 84A-3156, Mine Safety Appliance Company) showed lowest fit test rate [41]. N95 respirators are certified under NIOSH 42 CFR 84 regulations after passing the tests performed using charge-neutralized sodium chloride aerosol with the particle size of approximately 0.3 µm or 300 nm in diameter [42]. The certification criterion for N95 half-facepiece respirators says that the total momentary particle penetration (P = Concentration inside mask/Concentration outside mask × 100) through the respirator filter cannot exceed 5% at 85 L/min, i.e., the filtration efficiency, defined as E = 100% − P, must be at least 95%. Therefore, we conducted our experiments at a simulated inhalation air flow rate of 85 L/min. Respiratory protection devices with less efficient filtration characteristics are not NIOSH-certified. The value of 0.3 µm or 300 nm is presently accepted as the most penetrating particle size (MPPS) and fraction for various particulate filters. However, MPPS can vary substantially from one filter type to another and is dependent on the conditions at work sites. The data collected by Brown [43] indicated that the MPPS may be as high as 700 nm when very low (0.001 m/s) air velocity is passing through the filter. The MPPS may also depend on the fiber charge for pre-treated respirator filter media [44]. Most of these experiments on MPPS, however, were laboratory-based experiments and real-time data in construction work environments are inadequate.

**Figure 3:**
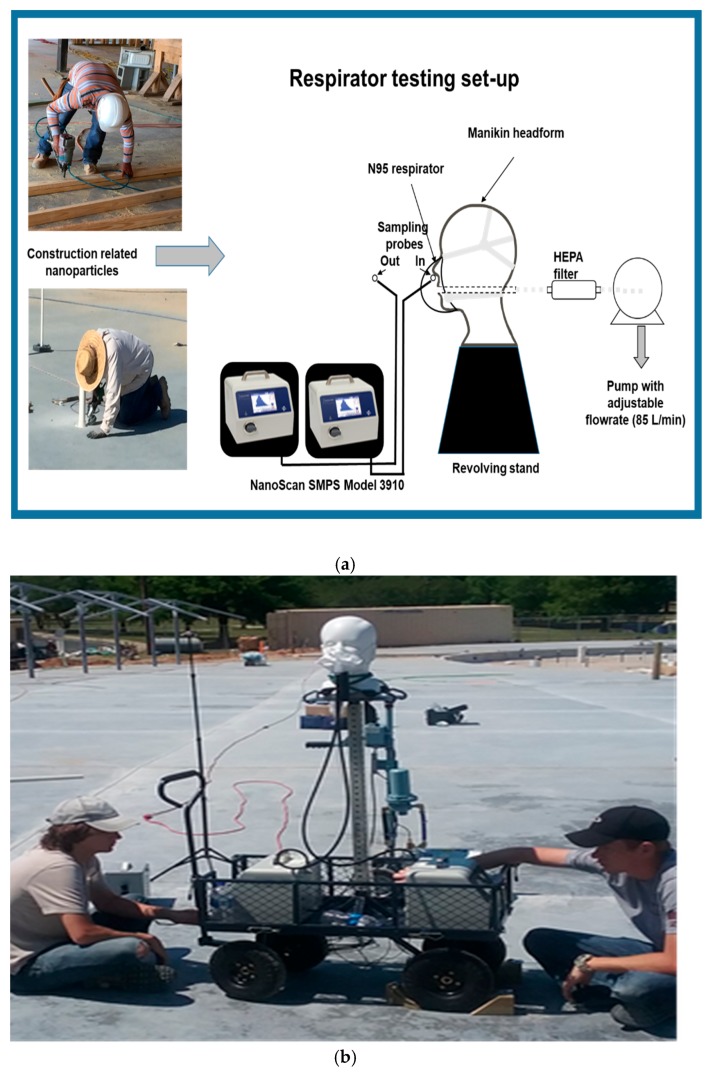
(**a**) Schematic of the respirator evaluation system for assessing protection offered by N95 filtering facepiece respirators in construction jobsites. (**b**) Photograph of the manikin-based N95 respirator evaluation set-up in a concrete blasting construction job site.

The N95 particulate respirators were placed on manikin faces and sealed so that no leakage occurred on the faceseal between the face and the inner lining of mask surfaces (Figure 3). We concurrently collected ultrafine particle samples from inside and outside of the respirator masks and particle penetration percentages were calculated (see below the results section) for 13 particle sizes: 11.5, 15.4, 20.5, 27.4, 36.5, 48.7, 64.9, 86.6, 115.5, 154, 205.4, 273.8, and 365.2 nm (median sizes of the particles collected in 13 collection bins). Ten samples were collected for each experiment. We have conducted some experiments with two leakage probes which were mounted on respirator surfaces, simulating potential faceseal leakages expected during actual work. However, these probes were probably partially blocked in outdoor dusty environments at construction jobsites and we did not receive consistent data. Therefore, this part of the study is not included in this article. We also excluded presenting penetration data for 205.4, 273.8, and 365.2 nm particles because occasionally some bins for these size ranges showed inconsistent values and zero readings due to some mechanical problems in the monitors.

### 2.3. Experiments with Charge Neutralized N95 Respirators

Because the filtration mechanism for N95 filtering facepiece respirators relies heavily on electrical charges, if large particles in ambient air in construction sites are charged and affecting the charge of the respirators, this could definitely affect the capture of ultrafine particles. To better understand the interaction between filtration and leakage processes without interference from electrostatic charge, we also conducted a few tests (*n* = 2 × 3 = 6) where respiratory protection against ultrafine particles was tested for N95 masks that had their electrostatic charge removed by isopropanol immersion, as described previously [36]. In brief, The N95 respirator masks were dipped into isopropanol for one min, removed, and then allowed to dry overnight in a clean chemical safety hood. This part has been included because we expected that removal of electrostatic charge from the filter media of the N95 respirator masks will shift the maximum penetrating particle size toward larger sizes, as observed previously in the laboratory study of NIOSH researchers [36].

### 2.4. Data Analysis

To examine differences between ultrafine particle concentration levels with reference to inside and outside sampling in N95 respirator masks and between different work tasks in each construction site, independent sample *t*-tests were conducted for normally distributed data. Q-Q plots and Shapiro-Wilk tests were used to check the normal distribution of data. Independent sample ANOVA tests were conducted for understanding the differences between penetrations of different categories of particles. Post hoc Scheffe tests were conducted to understand which specific particle sizes have significantly different penetration compared to all other test particles. All statistical analyses were performed using SPSS 11.0 for Windows (SPSS Inc., Chicago, IL, USA) and SAS/Stat 9.1 (SAS Institute Inc., Cary, NC, USA).

## 3. Results

**Figure 4:**
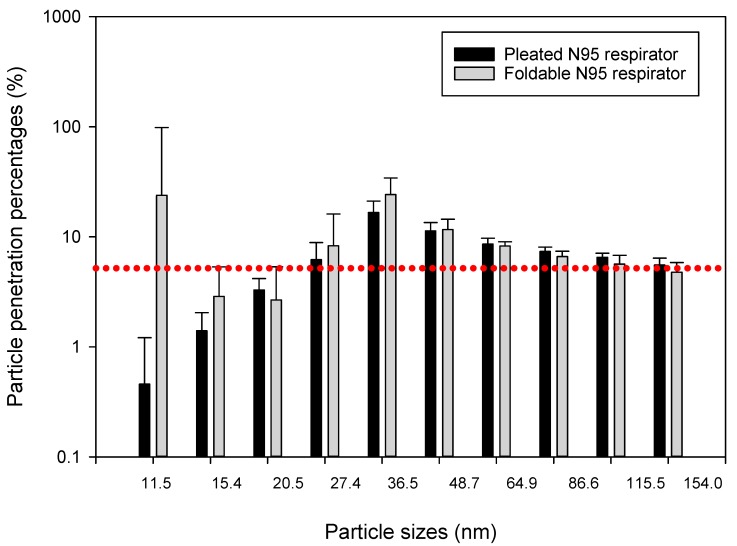
Penetration percentages of particles of 11.4–154.0 nm size range during concrete blasting and grinding. The dotted red line indicates the 5% particle penetration percentages, to be considered as the efficacy threshold for N95 respirators.

**Figure 5:**
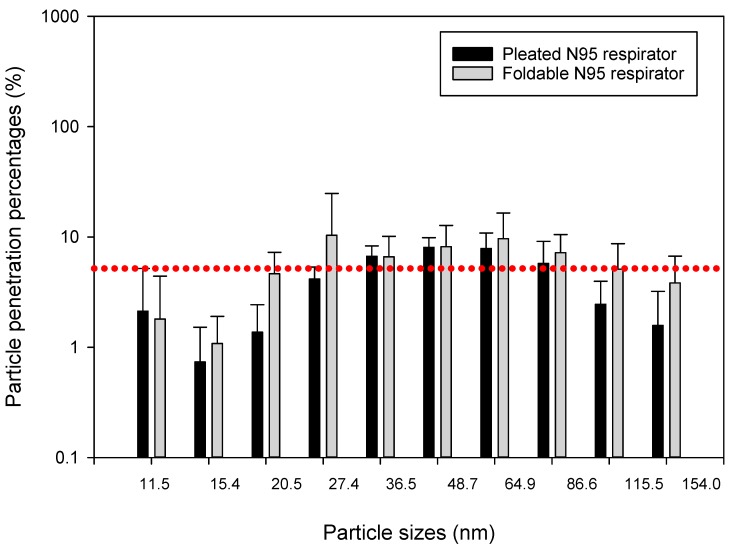
Penetration percentages of particles of 11.4–154.0 nm size range during wooden building frameworks construction. The dotted red line indicates the 5% particle penetration percentages, to be considered as the efficacy threshold for N95 respirators.

**Figure 6:**
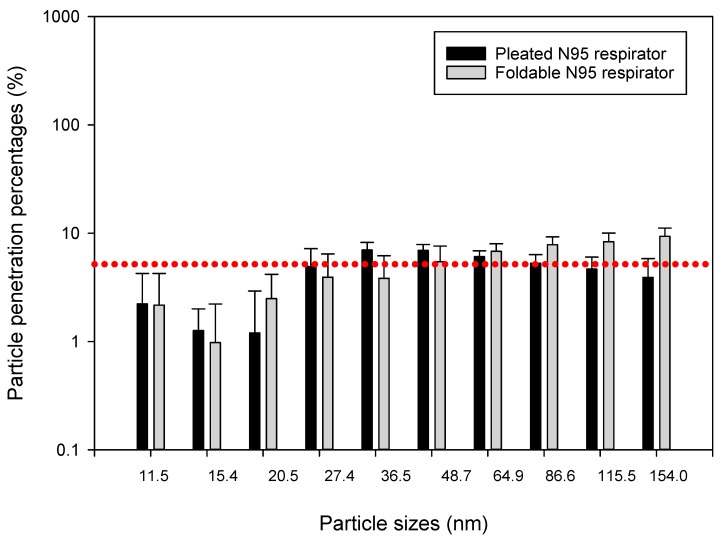
Penetration percentages of particles of 11.4–154.0 nm size range during soil moving by bulldozers in a large construction site. The dotted red line indicates the 5% particle penetration percentages, to be considered as the efficacy threshold for N95 respirators.

Data collected showed that different construction tasks can release different levels of airborne ultrafine particles ranging from 10^3^ to 10^5^ particles/cm^3^ at the surface of the tested respirator masks, which were placed about 5 to 10 m away from the actual construction activity. We compared particle penetration percentages between pleated and foldable N95 respirators by independent sample *t*-tests (Q-Q plots and Shapiro-Wilk test outputs showed that most datasets were normally distributed). Mean (±SD) penetration percentages for particles collected in 10 size bins of the NanoScan monitors are presented through bar charts and SD error bars in Figure 4, Figure 5 and Figure 6. We found that penetration of particles differs according to particle sizes, as well as type of construction activity. For example, in the concrete blasting/grinding site we found greater penetration of 36.5 nm particles in foldable mask (*p* < 0.05) than pleated mask, whereas 86.6 and 115.5 nm particles penetrated more (*p* < 0.05) in the pleated mask. Particles sized 20.5, 115.5, and 154.0 nm penetrated more (*p* < 0.05) in foldable mask in the wooden building frameworks construction site, whereas 36.5, 86.6, 115.5, and 154.0 nm particles penetrated more (*p* < 0.05) in foldable mask in soil moving site. Upon isopropanol treatment, significantly higher (*p* < 0.05) penetrations were observed for pleated masks than foldable masks (20.5, 27.4, and 36.5 nm particles in the wooden building frameworks construction site and 48.7, 64.9, and 154.0 nm particles in soil moving site; concrete blasting site was not tested for this experiment.

**Figure 7:**
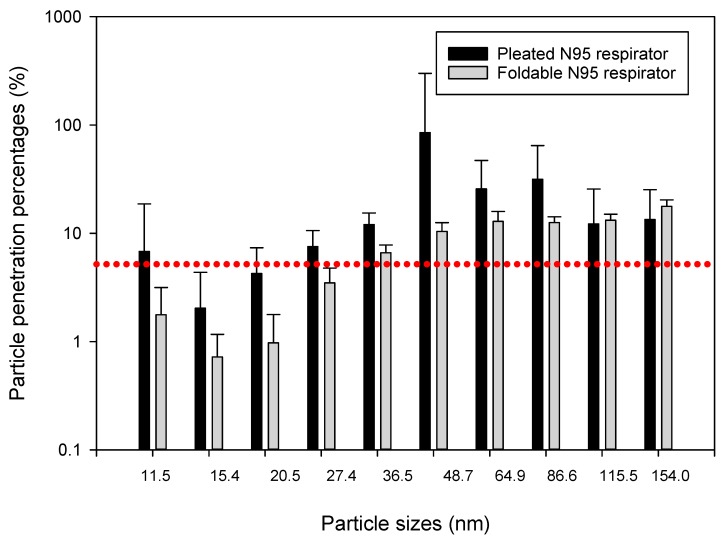
Penetration percentages of particles of 11.4–154.0 nm size range in isopropanol treated N95 masks during wooden building frameworks construction. The dotted red line indicates the 5% particle penetration percentages, to be considered as the efficacy threshold for N95 respirators.

**Figure 8:**
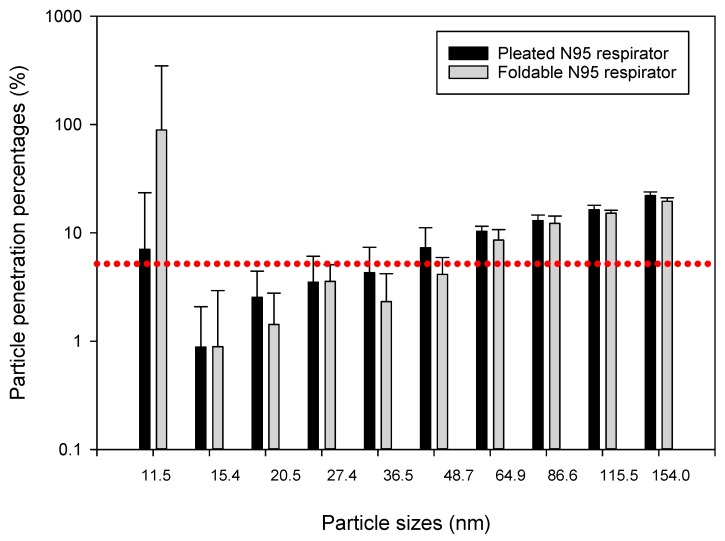
Penetration percentages of particles of 11.4–154.0 nm size range in isopropanol-treated N95 masks during soil moving in a large construction site. The dotted red line indicates the 5% particle penetration percentages, to be considered as the efficacy threshold for N95 respirators.

Previously we compared penetration of the same particles into two types of masks—pleated and foldable. In this section, we are presenting the differences between penetration percentages of particles of different sizes. Because individual particle penetration datasets were mostly normally distributed, we conducted independent sample ANOVAs for understanding the differences between penetrations of different categories of particles in the same mask. Except for the foldable mask in the concrete blasting/grinding site, all other comparisons showed significant differences between penetration percent levels for different particles (*p* < 0.05). In post hoc Scheffe tests, particles subset of 27.4, 36.5, 48.7, 64.9, and 86.6 nm showed significantly different (*p* < 0.05) penetration than other particles in the wooden building frameworks construction site for pleated masks, whereas particles subset of 11.5, 27.5, 86.6, 115.5, and 154.0 nm sizes showed significantly different (*p* < 0.05) penetration trend from other particles in soil moving site in pleated masks. No such significant trends were observed for foldable masks in these two sites, nor for both pleated and foldable masks in the concrete blasting/grinding site. Interestingly, similar trends were not observed in the isopropanol-treated pleated and foldable masks when post hoc Scheffe tests were conducted (Figure 7 and Figure 8).

Temperature and relative humidity levels of three construction sites were monitored during the field experiments. Ten readings were collected from each site using a portable thermohygrometer connected to a handheld particle counter. Average temperature (mean ± SD) were 29.62 ± 0.65 °C, 24.57 ± 0.11 °C, and 25.87 ± 0.56 °C, respectively at concrete blasting, wooden building construction, and soil moving sites. Relative humidity levels in these three sites were 36.62 ± 0.73%, 65.5 ± 0.33%, and 58.04 ± 1.36%, respectively. These data indicate that relative humidity level at the concrete blasting site was lower than the other two sites. Low standard deviations in the data indicate that the meteorological conditions were relatively stable during the field experiments.

## 4. Discussion

This study addresses the need in methods and techniques for the quantification of respiratory protection levels against ultrafine particles on various construction jobsites offered by N95 particulate respirators. Most of the previous studies on nanoparticle penetration through NIOSH-approved N95 masks were conducted in laboratory conditions and to our knowledge, this is the first study conducted in construction jobsites addressing this topic. Previous researchers reported that the most penetrating particle size was found to be near 40 nm in a laboratory study using polydisperse aerosol, and average penetration of 40 nm particles was found to exceed 5% for two N95 respirator models [45]. We found similar trends in concrete grinding and blasting site for 36.5 nm particles, but not in wooden building construction sites and soil moving sites. Therefore, the physical properties and electrostatic charges of ultrafine particles might be different when they have originated from different sources, and these properties may influence penetrations through charged N95 respirator fibers. We found that after charge removal, penetration of ultrafine particles of medium to large size ranges (mostly >50 nm) increased.

One common problem with conducting experiments at construction jobsites was that in some cases, the managers were reluctant to provide access to the jobsite to research team members due to potential safety hazards and liability issues. One other unexpected issue was the inconsistency between the results obtained from two different Nanoscans used, which we described above. The scientific data generated in this exploratory study and challenges identified will advance the knowledge about respiratory protection against airborne ultrafine particles in various construction sites. Our data will also provide new information on most penetrating ultrafine particle sizes through N95 masks in field conditions (construction sites), which may be relevant for NIOSH, OSHA, and ACGIH for future research and recommendations on appropriate respiratory protection against ultrafine particles. The unique findings in this study and few challenges are believed to be useful for workers’ safety and health issues pertinent to ultrafine particle exposures and establishing the linkage between exposure to ultrafine particles in work environments and potential respiratory and inflammatory health effects among construction workers. 

We found that penetration of ultrafine particles differed with respect to particle size, as well as the type of N95 masks. N95 respirators may not provide 95% protection for all categories of ultrafine particles, including the subset of nanoparticles. We found that ultrafine particles of <36.5 nm size generally penetrated least through both types of pleated and foldable respirator models when compared with other particle sizes (except a few exceptions for 11.5 nm particles).

The findings described above underscore the need for improving construction workers’ respiratory protection against ultrafine particles during different construction related activities involving concrete, wood, and soil cutting, grinding, handling, and moving. Besides engineering controls and best work practices, adequate respiratory protection is recommended to prevent exposure to ultrafine particles, including incidental nanoparticles released from these various tasks. Certainly, we need more research in this area.

## 5. Conclusions

In conclusion, we found that foldable N95 filtering facepiece respirators were less efficient than pleated N95 respirators in filtering ultrafine particles mostly at the soil moving site and the wooden building frameworks construction site. Filtration efficiency of foldable N95 masks was generally lower for particles of tested medium ultrafine size ranges. Filtration efficiencies of N95 masks differed in different construction operations, likely due to different environmental conditions and/or physical properties of ultrafine particles. Upon isopropanol treatment, the particles of larger sizes penetrated more compared to particles of smaller sizes.
